# Effects of multiwalled carbon nanotubes and triclocarban on several eukaryotic cell lines: elucidating cytotoxicity, endocrine disruption, and reactive oxygen species generation

**DOI:** 10.1186/1556-276X-9-396

**Published:** 2014-08-15

**Authors:** Anne Simon, Sibylle X Maletz, Henner Hollert, Andreas Schäffer, Hanna M Maes

**Affiliations:** 1 Institute for Environmental Research (Biology V), RWTH Aachen University, Worringerweg 1, Aachen 52074, Germany; 2 School of Environment, Nanjing University, Nanjing 210023, China; 3 Key Laboratory of Yangtze River Environment of Education Ministry of China, College of Environmental Science and Engineering, Tongji University, Shanghai 200092, China; 4 College of Resources and Environmental Science, Chongqing University, Chongqing 400715, China

**Keywords:** Cytotoxicity, Endocrine disruption, Multiwalled carbon nanotubes, Nanotoxicology, Oxidative stress, Triclocarban

## Abstract

To date, only a few reports about studies on toxic effects of carbon nanotubes (CNT) are available, and their results are often controversial. Three different cell lines (rainbow trout liver cells (RTL-W1), human adrenocortical carcinoma cells (T47Dluc), and human adrenocarcinoma cells (H295R)) were exposed to multiwalled carbon nanotubes, the antimicrobial agent triclocarban (TCC) as well as the mixture of both substances in a concentration range of 3.13 to 50 mg CNT/L, 31.25 to 500 μg TCC/L, and 3.13 to 50 mg CNT/L + 1% TCC (percentage relative to carbon nanotubes concentration), respectively. Triclocarban is a high-production volume chemical that is widely used as an antimicrobial compound and is known for its toxicity, hydrophobicity, endocrine disruption, bioaccumulation potential, and environmental persistence. Carbon nanotubes are known to interact with hydrophobic organic compounds. Therefore, triclocarban was selected as a model substance to examine mixture toxicity in this study. The influence of multiwalled carbon nanotubes and triclocarban on various toxicological endpoints was specified: neither cytotoxicity nor endocrine disruption could be observed after exposure of the three cell lines to carbon nanotubes, but the nanomaterial caused intracellular generation of reactive oxygen species in all cell types. For TCC on the other hand, cell vitality of 80% could be observed at a concentration of 2.1 mg/L for treated RTL-W1 cells. A decrease of luciferase activity in the ER Calux assay at a triclocarban concentration of 125 μg/L and higher was observed. This effect was less pronounced when multiwalled carbon nanotubes were present in the medium. Taken together, these results demonstrate that multiwalled carbon nanotubes induce the production of reactive oxygen species in RTL-W1, T47Dluc, and H295R cells, reveal no cytotoxicity, and reduce the bioavailability and toxicity of the biocide triclocarban.

## Background

The annual worldwide production of carbon nanotubes (CNT) surpassed the multimetric ton level and is expected to further increase [1]. Their structure gives them exceptional properties, which makes this material suitable for the use in composite materials, sensors, drug delivery, hydrogen storage fuel cells, and various environmental applications [2-4]. The probability of occupational and public exposure to CNT has significantly increased [5]. With this nanophase invasion of new materials and products into many aspects of life comes the need for increasing safety measures for exposure risks [6]. In October 2011, the European Union defined nanomaterials as natural, incidental, or manufactured materials containing particles, in an unbound state or as an aggregate or agglomerate, where 50% or more of the particles exhibited one or more external dimension in the size range of 1 to 100 nm [7]. Carbon nanotubes represent one of the most promising nanomaterials for various applications [8]. However, public concerns on the widespread use of these materials increase due to their close similarity to other toxic fibers regarding their high aspect ratio, reactivity, and biopersistence. Multiwalled carbon nanotubes (MWCNT) used in this study were the most highly produced CNT materials until 2012 [8]. A pilot plant with an annual capacity of 60 tons is since 2007 in an operation in southern Germany. Thus, knowledge on the toxic potential of MWCNT is required also regarding the very different nature of various types differing in flexibility or stiffness, varying in length and aspect ratio as well as having different contents of metal catalysts and surface properties. All MWCNT have a tubular structure with a high aspect ratio and between 2 and 30 concentric cylinders with outer diameters commonly between 30 and 50 nm. The small size and the high surface area define the chemical reactivity of CNT and induce changes in permeability or conductivity of biological membranes [9]. Therefore, engineered CNT may pose health risks because of their ability to reach every part of the organs and tissues and their interaction with cellular functions. The primary risk of these materials may come from their ability to enter cells, which may cause damage to plants, animals, and humans [10-13]. Important characteristics are the surface chemistry and purity of CNT. For MWCNT synthesized using a metal catalyst, the toxicity may be the combined effect of the MWCNT themselves and an oxidative stress response to the residual metal catalyst [14] typically amounting to less than about 5 wt.%. This complicates clear determination of pure MWCNT toxicity. Despite these concerns, very few studies have been simultaneously conducted with various human cell lines to assess the health effects of different CNT. At present, there is no global agreement about the risk of CNT on human health [15].

Previous researchers have explored the toxicity of carbon nanomaterials to animal and human cells [16-20]. It was suggested that the toxicity of carbon nanomaterials may also be caused by sorption of toxic substances to their surface [21-23]. Therefore, knowledge of toxic compound adsorption by carbon nanomaterials is critical and useful for risk assessment of these nanomaterials because in the environment, both nanomaterials and chemical pollutants, are present as complex mixtures.

CNT are carbonaceous adsorbents with hydrophobic surfaces that exhibit strong adsorption affinities to organic compounds [24-30]. Thereby, a combination of chemical and physical interactions play a major role for adsorption processes. CNT have uniform structural units but are prone to aggregate, forming bundles of randomly tangled agglomerates because of the strong van der Waals forces along the length axis [31]. The outermost surface, inner cavities, interstitial channels, and peripheral grooves of CNT constitute four possible sorption sites for organic compounds [30]. Nanotechnology has initiated different types of nanomaterials to be used in water technology in recent years that can have promising outcomes. Nanosorbents such as CNT have exceptional adsorption properties and can be applied for removal of heavy metals, organics, and biological impurities [28,32]. CNT, as adsorbent media, are able to remove heavy metals such as Cr^3+^[33], Pb^2+^[34], and Zn^2+^[35], metalloids such as arsenic compounds [36], organics such as polycyclic aromatic organic compounds (PAH) [24,29], pesticides [37], and a range of biological contaminants including bacteria [38-40], viruses [41,42], cyanobacterial toxins [43,44] as well as natural organic matter (NOM) [45-47]. The success of CNT as an adsorbent media in the removal of biological contaminants, especially pathogens is mainly attributed to their unique physical, cytotoxic, and surface functionalizing properties [28].

To date, many studies on the safety of different CNT materials have been conducted but the results are often controversial and depending of the species of the applied CNT. A wide range of results from in vitro studies, dealing with MWCNT, has been reported. On the one hand, MWCNT decreased cell viability and induced apoptosis [48,49], whereas minimal to no decrease of cell viability was observed [50]. One explanation of this controversy is the type of cells used. Additional explanations are that MWCNT are produced by different processes, tested with varying dispersion methods, and that their life cycle may confer changes in their surface characteristics and reactivity. For example, in some studies, the presence of metal trace impurities explains demonstrated toxicity and reactive oxygen species (ROS) production [50], whereas in other cases, no such effects were reported [51]. Nevertheless, it is recognized that nanoparticles produce ROS [50,52] inside and outside the cell, which has to be considered as one of the key factors for toxicological effects [6]. Hence, further evaluation and characterization of their toxic potential and other effects on cells like cytotoxicity, endocrine disruption, and the production of ROS, which can result in cell damage, is of highest concern.

Relatively little research has been conducted examining biocidal components of personal care products, as for example triclocarban (TCC), although such products are continually released into the aquatic environment and are biologically active and some of them persistent [53]. Therefore, they are detected often and in rather high concentrations in the environment [53]. TCC is a high-production volume chemical [54] that is widely used as an antimicrobial compound [53,55]. It is able to adsorb on the cell membrane and to destroy its semi-permeable character, leading to cell death [56]. In the U.S., the annual production of TCC in 2002 added up to 500 metric tons [57,58]. The primary route for TCC to enter the environment is through discharge of effluent from wastewater treatment plants and disposal of solid residuals on land [55,58]. Due to its lipophilicity (log Kow 4.9 [59]), TCC has an affinity to adsorb to organic matter [60]; therefore, over 70% of the initial mass has been found to be adsorbed to sludge [61,62]. TCC has been detected at microgram per liter levels in waterways in the United States and Switzerland, indicating extensive contamination of aquatic ecosystems [54,63,64]. TCC was chosen in this study for its widespread use, toxicity [58], bioaccumulation potential [65,66], environmental persistence, and endocrine effects [67].

As TCC is used since 1957 in huge amounts [53], and MWCNT is supposed to reach the amount of a large scale production, both substances might involuntarily occur together in the environment.

This study aimed to provide new information on toxicity of TCC and nanotoxicity of MWCNT as well as the mixture of both substances by using three different eukaryotic cell lines. Key questions were to get more information about the cytotoxicity of MWCNT and the estrogenic potential of TCC as well as the potential of MWCNT to generate ROS in cell lines. Especially, the interaction of MWCNT and TCC poses a major question in the present study, if one of them is more or less toxic when cells are exposed to mixtures of both.

As many studies already showed that CNT are toxic for different cell lines [5,9], we investigated cells by determination of cytotoxicity in the neutral red retention (NR) assay and the 3-(4,5-dimethylthiazol-2-yl)-2,5-diphenyl tetrazolium bromide (MTT) assay [68] to verify whether MWCNT showed a toxic potential for the used cells, namely RTL-W1, T47Dluc, and H295R. A combination of cytotoxicity assays, particularly the NR and MTT assay, was preferred in many studies [69-71], as this would increase the reliability of the results obtained.

Furthermore, mechanism-specific endpoints, such as estrogenic effects and alterations of the steroid synthesis were analyzed by using the estrogen receptor-mediated chemical-activated luciferase gene expression (ER-Calux) assay [72] and the H295R steroidogenesis assay (H295R) [73,74], respectively. The evaluation of the endocrine activity in wastewater samples could already been proven by using these assays [75-78]. As previously reviewed by Hecker and Hollert [79], results of several studies indicated that a combined use of receptor-mediated and non-receptor-mediated methods is necessary to enable objective assessment of endocrine potential in complex samples. Additionally, Grund et al. [80] demonstrated that the combination of receptor-mediated and non-receptor-mediated assays such as the ER Calux and the H295R was appropriate for a holistic evaluation of potential endocrine activity of complex environmental samples.

The measurement of cellular reactive oxygen species was investigated by using the fluorescent dye 2′,7′-dichlorodihydrofluorescein diacetate (H_2_DCF-DA) assay [81].

## Methods

### Chemicals

The test substance 3,4,4′-trichlorocarbanilide was purchased from Sigma Aldrich (St. Louis, MO, USA) and had a purity of 99% (CAS:101-20-2). Multiwalled carbon nanotubes (Baytubes C150P, >95% purity) were provided from Bayer MaterialScience (Bayer AG, Leverkusen, Germany). The used concentrations of both materials in the different test systems were based on limit tests and not higher than the dispersibility of CNT or solubility of TCC.

### Cell cultures

#### RTL-W1 cells

The rainbow trout liver cell line (RTL-W1) [82] was grown in L15-Leibovitz medium (Sigma-Aldrich) supplemented with 9% fetal bovine serum (FBS, Biowest, Logan, UT, USA) and penicillin/streptomycin (10,000 E/mL; 10,000 μg/mL in 0.9% NaCl, Sigma-Aldrich) in 75-cm^2^ flasks (Techno Plastic Products (TPP), Trasadingen, Switzerland) at 20°C in darkness according to the protocol detailed in Klee et al. [83].

#### T47Dluc cells

The human T47Dluc breast adenocarcinoma cells were obtained from BioDetection Systems BV (Amsterdam, the Netherlands) and were cultured in Dulbecco's modified Eagle medium/nutrient mixture F-12 (DMEM/F12) with phenol red (Gibco, Grand Island, NY, USA) supplemented with sodium bicarbonate (Sigma-Aldrich), MEM 100× (Gibco), penicillin/streptomycin solutions (Gibco) and 7.5% fetal bovine serum (FBS) according to the methods details in Maletz et al. [84]. T47Dluc cells were cultured at 37°C, 7.5% CO_2_, and maximum humidity.

#### H295R cells

The human adrenocarcinoma cells (H295R) were obtained from the American Type Culture Collection (ATCC; Manassas, VA, USA) and were grown in 75-cm^2^ flasks with 8 mL supplemented medium at 37°C with a 5% CO_2_ atmosphere as described previously [73,85].

### Nanoparticles suspension

**Figure 1:**
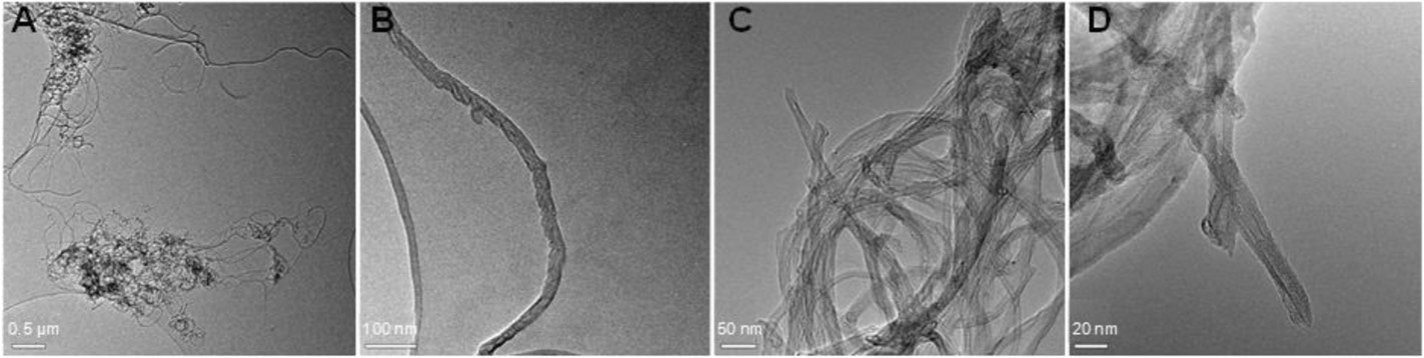
**TEM pictures of MWCNT.** Agglomerates **(A)**, single nanotubes **(B)**, and tubes sticking out of the agglomerates **(C, D)** visualized by transmission electron micrographs of sonicated MWCNT in distilled water.

Test suspensions of 1 to 100 mg/L of MWCNT were prepared by ultrasonication of the raw material with a microtip (70 W, 0.2″ pulse and 0.8″ pause; Bandelin, Berlin, Germany) in distilled water for 10 min. Transmission electron microscopy (TEM) images showed the presence of small agglomerates and individual nanotubes in the medium (Figure 1).

### Cytotoxicity assays

For determining the effect of particles on cell viability, different assays were used. Potential interferences of MWCNT and the fluorescence measurement were prevented by using black microtiter plates.

### Neutral red retention assay

The neutral red retention (NR) assay was performed according to Borenfreund and Puerner [86] with slight modifications as detailed in Heger et al. [87] by using RTL-W1 cells. Briefly, 4 × 10^5^ cells were seeded into each well (except for the blanks) of a 96-well microtiter plate (Nunc) and directly treated in triplicates with the particle suspensions. To guarantee optimal culture conditions, cells were exposed in a 1:1 mixture of MWCNT suspension or TCC solution and double-concentrated L15-Leibovitz medium, resulting in final MWCNT-concentrations of 3.13 to 50 mg CNT/L and TCC concentrations of 7.8 to 10 × 10^3^ mg/L. After incubation for 48 h at 20°C in the dark, the sample solution was discarded, and each well was rinsed with 100 μL phosphate-buffered saline (PBS) to remove any excess medium. One hundred microliters of a 0.005% neutral red solution (2-methyl-3-amino-7-dimethylaminophenanzine, Sigma-Aldrich) was added to each well except for the blanks. After an incubation time of 3 h at 20°C in darkness, the amount of extracted NR was determined by absorption measurement at 540 nm and a reference wavelength of 690 nm using a microtiter plate reader (Infinite M200, Tecan Instruments, Männedorf, Switzerland). Thereafter, concentrations resulting in cell vitality of 80% were calculated and identified as NR_80_ values according to Heger et al. 2012 [87]. For detection of significant differences, the *t test* following square root transformation was performed using SigmaPlot 12. Results are given as relative values to the untreated control in percent.

### MTT assay

The cell viability was evaluated by the reduction of water soluble 3-(4,5-dimethylthiazol-2-yl)-2,5-diphenyl tetrazolium bromide (MTT, Sigma Aldrich) to water-insoluble formazan crystals by mitochondrial dehydrogenase [88]. The amount of the formed blue formazan is proportional to the amount of viable cells [89], and the absorbance was measured at 492 nm using a microtiter plate reader (Tecan).

### H295R cells

The exposure of H295R cells was conducted according to the methods of Hecker et al. [73,74]. In brief, 1 mL of cell suspension, at a concentration of 2.5 × 10^5^ H295R cells/mL, was added to each well of a 24-well microtiter plate and cells were allowed to attach for 24 h. Cells were treated in triplicate with a 1:1 mixture of the MWCNT suspension and/or TCC solution and double-concentrated medium, resulting in final concentrations of 3.13 to 50 mg CNT/L and 31.25 to 500 μg TCC/L for 48 h as well as the two reference substances forskolin and prochloraz (quality control plate). The plates were checked for cytotoxicity and contamination after 24 h of exposure. The culture supernatants were removed and frozen at -80°C for later analysis of alterations in steroid synthesis in the enzyme-linked immunosorbent assay (ELISA) assay. Cells were rinsed with 600 μL PBS per well. Then, 400 μL of a freshly prepared MTT (thiazolyl blue tetrazolium bromide, ≥ 97.5% TLC) solution at 500 μg/mL was added to each well and incubated for 30 min at 37°C and 5% CO_2_ in air atmosphere. The MTT solution was discarded, and 800 μL DMSO was added to each well in order to lyse the cells. Plates were finally placed on a horizontal shaker for 10 to 15 min before measuring the absorbance. Results are given as relative values to the solvent control in percent.

### T47Dluc cells

The MTT assay was performed according to Mosmann [90]. In brief, T47Dluc cells were seeded into a 96-well microtiter plate (TPP) at a density of 1 × 10^4^ cells per well. After 24 h of pre-incubation, the old medium was removed and cells were treated with a 1:1 mixture of the MWCNT suspension and/or TCC solution and double-concentrated medium. A serial dilution resulted in five concentrations of the MWCNT suspension and TCC solution and a solvent control were applied to each plate. For each concentration, three wells were foreseen. The exposure medium was removed, and the absorbance was measured after adding the freshly prepared MTT solution (500 μg/mL, Sigma-Aldrich) with a luminescence counter (Tecan) at 492 nm.

For both cell lines (H295R and T47Dluc), concentration-response curves were fitted with a non-linear ’log(agonist) vs. response - variable slope’ regression using GraphPad Prism 5 as detailed in Heger et al. [87].

### ER Calux

The ER Calux assay with stably transfected T47Dluc human breast cancer cells was developed by Legler et al. [72] and was conducted in this study according to the detailed protocol given in Maletz et al. [84]. T47Dluc cells/mL (10 × 10^4^), resulting in a density of 1 × 10^4^ cells per well, were plated into 96-well microtiter plates in medium (DMEM/F12 free of phenol red supplemented with sodium bicarbonate, MEM 100×, and fetal calf serum) and incubated for 24 h at 37°C (7.5% CO_2_, 100% humidity). After this time, the assay medium was renewed, and the cells were incubated for another 24 h. Then, a 1:1 mixture of the MWCNT suspension and/or TCC solution and double-concentrated medium replaced the medium by using a serial dilution resulting in five concentrations. All concentrations of the test compound and the positive control (E2) as well as blanks (DMSO) and solvent control (EtOH) were introduced to each plate in triplicate. After 24 h of exposure, the plates were checked for cytotoxicity and contamination and the medium was removed. Following the addition of a mixture of 1:1 of PBS and steady light solution (PerkinElmer Inc., Waltham, MA, USA), the plates were incubated on an orbital shaker in darkness for 15 min. Luminescence was measured using a plate reader (Tecan). The luciferase activity per well was measured as relative light units (RLU). The mean RLU of blank wells was subtracted from all values to correct for the background signal. The relative response of all wells was calculated as the percentage of the maximal luciferase induction determined for E2 [91]. Only suspensions that did not cause cytotoxicity were used for quantification of the response.

### Enzyme-linked immunosorbent assay

For quantification of hormone production by H295R cells, the protocol given by Hecker et al. [73,74] was used. To ensure that modulations in hormone synthesis were not a result of cytotoxic effects, viability of the cells was assessed with the MTT bioassay [90] before initiation of exposure experiments. Only non-cytotoxic concentrations (>80% viable cells per well) were evaluated regarding their potential to affect steroid genesis [80]. In brief, H295R cells were exposed as described above. The frozen medium was thawed and extracted using liquid extraction with diethylether as described previously in Maletz et al. [84]. The amount of 17β-estradiol (E2) was determined in an enzyme-linked immunosorbent assay (ELISA) assay (Cayman Chemicals, Ann Arbor, MI, USA) [80].

### Measurement of cellular ROS

The production of reactive oxygen species in RTL-W1, T47Dluc, and H295R cells were measured using the fluorescent dye 2′,7′-dichlorodihydrofluorescein diacetate (H_2_DCF-DA) as previously described [50,81,92-95]. This dye is a stable cell-permeant indicator which becomes fluorescent when cleaved by intracellular esterases and oxidized by intracellular hydroxyl radical, peroxynitrite, and nitric oxide [92]. The intensity of fluorescence is therefore proportional to the amount of reactive oxygen species produced in cells. RTL-W1, T47Dluc, and H295R cells were charged as explained above, except for that H295R cells were seeded in 96-well plates as well. After an exposure time of 24 or 48 h, the medium was discarded, cells were washed three times with PBS because black particles strongly reduced the fluorescence signal, and 100 μL of H_2_DCF-DA (final concentration of 5 μM in PBS) was added to each well. Subsequently, the plates were incubated for 45 min at room temperature on a horizontal shaker in darkness. Fluorescence at excitation and emission wavelengths of 485 and 530 nm, respectively, was measured with a microtiter plate reader (Tecan).

### Statistical methods

Statistical analyses were carried out with SigmaPlot 12. Results are presented as mean ± standard deviation (SD). To enhance the comparability of the assays, the results were normalized to the average value of the solvent controls (SC) and are expressed as percent change or fold change relative to the SC. Prior to conducting statistical analyses, all data were checked for normality and homogeneity of variance using the Kolmogorov-Smirnov and Levene's test. A one-way analysis of variance (ANOVA) followed by Dunnett's *post hoc* test was used to determine treatments that differed significantly from the SC for data fulfilling the parametric assumptions. Otherwise, the non-parametric Kruskall-Wallis test followed by Dunn's *post hoc* test was used. For the detection of significant differences in cytotoxicity assays, the *t* test following square root transformation was performed. Differences were considered significant at *p* < 0.05.

## Results

### Cytotoxicity

#### Neutral red retention assay

**Figure 2:**
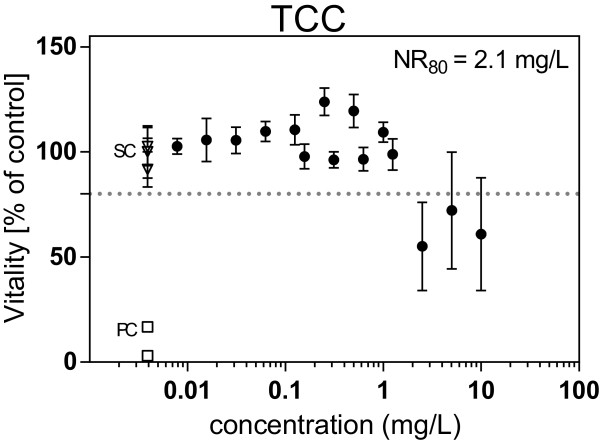
**Cytotoxic effects of TCC in the NR assay.** Cytotoxicity of TCC assessed in the neutral red retention assay with RTL-W1 cells. Dots represent the mean of three independent exposure experiments with three internal replicates and are given in percent of the viability of the control. The whiskers show the standard deviation of the mean; PC, positive control (3,5-dichlorophenol); SC, solvent control (EtOH); the dashed line marks the threshold of 80%.

An NR_80_ value (concentrations resulting in 80% viability of the RTL-W1 cells) of 2.1 mg/L was obtained for the biocide TCC (Figure 2). The exposure of cells to MWCNT at concentrations ranging between 0.78 and 50 mg/L and to the mixture of CNT and TCC (0.39 to 25 mg CNT/L +1% TCC; percentage relative to CNT concentration) did not result in cytotoxicity.

Concentrations of TCC in the subsequently ROS assay were kept below 0.5 mg/L, i.e., below the NR_80_ value of 2.1 mg/L.

#### MTT assay

In addition to the testing of RTL-W1 cells, cytotoxicity was assessed for T47Dluc cells and H295R cells in the MTT assay.

**Figure 3:**
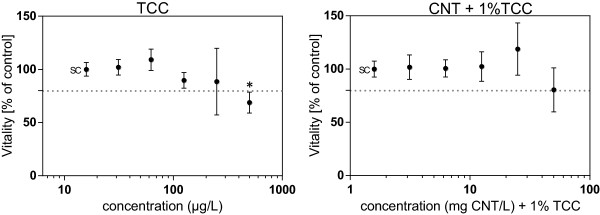
**Cytotoxicity of TCC and its mixture with CNT in the MTT assay with H295R cells.** Cytotoxicity of TCC and a mixture of CNT with 1% TCC (percentage relative to CNT concentration) as assessed in the MTT cell viability assay with H295R cells. Percent of viable cells after 48 h of exposure are given compared to the solvent control. Dots represent the mean of four independent exposure experiments with three internal replicates each. Error bars, standard deviation; SC, solvent control. The dashed line marks the threshold of 80%.

All concentrations of MWCNT (0.5 to 50 mg/L), TCC (31.25 to 500 μg/L), and the mixture of both substances (1.56 mg CNT/L + 15.6 μg TCC/L to 25 mg CNT/L + 250 μg TCC/L, i.e., CNT + 1% TCC) did not result in cytotoxicity in T47Dluc cells (data not shown).The results of the MTT cell viability assay with H295R cells are presented in Figure 3. The percentage of viable cells relative to the ethanol (EtOH) control is plotted against the respective sample concentration.The highest concentration of TCC (500 μg/L) revealed cytotoxicity after the exposure to H295R cells. In combination with CNT, lower cytotoxicity of the biocide was observed although the same concentration of TCC was applied to the cells (Figure 3). The lower cytotoxicity of the mixture testing was not significantly different from the exposure to TCC alone. MWCNT-treated cells showed no cytotoxicity after exposure to concentrations between 3.13 and 50 mg CNT/L (data not shown).

#### ER Calux assay

**Figure 4:**
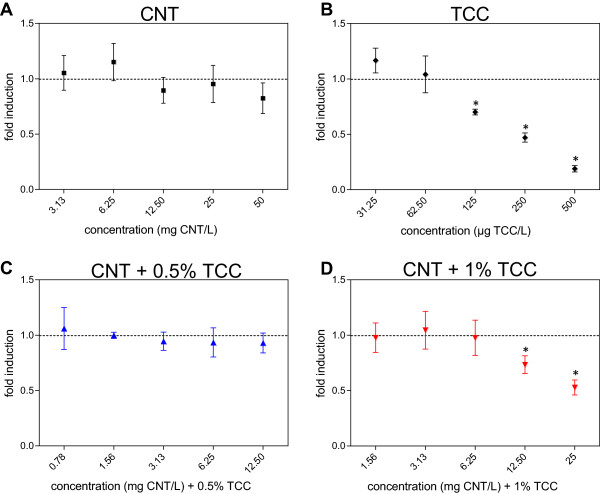
**Estrogenic disruption in the ER Calux assay with T47Dluc cells.** Estrogenic activity given as luciferase induction relative to solvent control (=1, dashed line) in the ER Calux assay plated in 96-well plates. T47Dluc cells were treated with CNT **(A)**, TCC **(B)**, and mixture of both (CNT + 0.5% TCC **(C)**, 1.56 mg CNT/L + 7.80 μg TCC/L to 25 mg CNT/L + 125 μg TCC/L; CNT + 1% TCC **(D)**, 1.56 mg CNT/L + 15.60 μg TCC/L to 25 mg CNT/L + 250 μg TCC/L). Dots represent means of two independent exposure experiments with three internal replicates each. Error bars, standard deviation; *statistically significant from the EtOH control in repeated measures ANOVA on Ranks with Dunn's *post hoc* and *p* < 0.05.

Estrogenic activities were determined in CNT suspensions, TCC dilutions, and mixture of both substances using the ER Calux assay. Figure 4A shows that CNT had no estrogenic effect in the range of 3.13 to 50 mg CNT/L. Interestingly, a decrease of luciferase activity by high concentrations of the biocide TCC can be seen in Figure 4B. Cytotoxicity could be excluded for the concentrations used as shown in the MTT assay with T47Dluc cells. The antiestrogenic potential of TCC was reduced when cells were exposed to the mixture of CNT and 0.5% TCC (Figure 4C). This effect was not observed after application of CNT including 1% TCC (Figure 4D).

#### Alterations of steroid synthesis in H295R cells

CNT did not have a pronounced effect on hormone production of 17β-estradiol (E2) in H295R cells. E2 levels were all in the range of the negative control. Also, after exposure to TCC concentrations, the hormones were at the level of the EtOH control. Mixture of CNT and TCC did not significantly alter production of E2 in H295R cells in the range of 1.56 mg CNT/L + 15.6 μg TCC/L to 25 mg CNT/L + 250 μg TCC/L.

### Measurement of cellular ROS

**Figure 5:**
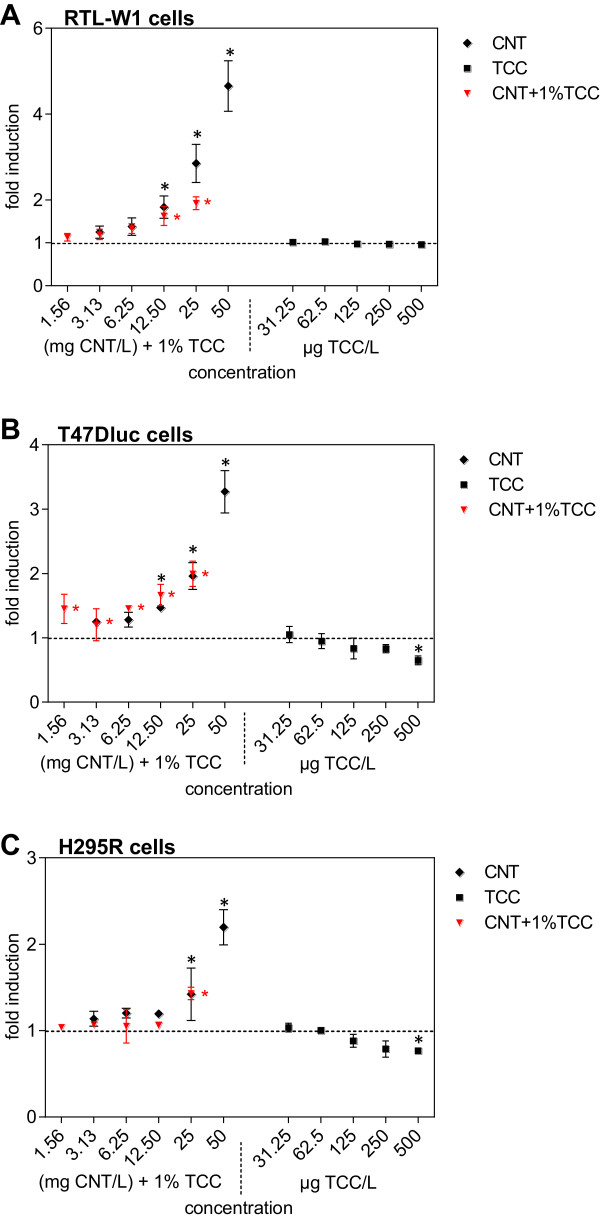
**Generation of ROS in RTL-W1, T47Dluc, and H295R cells.** ROS generated in RTL-W1 **(A)**, T47Dluc **(B),** and H295R **(C)** cells exposed to MWCNT, TCC, and mixture of both substances (1% TCC, with respect to the concentration of CNT). The intensity of H2DCF-DA was measured in cell lysates and normalized to negative/solvent control (=1, dashed line). Data are expressed as mean ± standard deviation of three independent exposure experiments with three internal replicates each. *Statistically significant from the negative control in repeated measures ANOVA on ranks with Dunn's *post hoc* and *p* < 0.05.

Effects of MWCNT and TCC on radical formation were assessed by measuring intracellular ROS in RTL-W1, T47Dluc, and H295R cells. Compared to the EtOH control, no significant difference in the ROS generation by TCC and the combination of MWCNT and TCC in all three cell lines was observed. In MWCNT-treated cells, however, a much higher ROS production than that in the controls was measured. The ROS content was 1.8, 2.9, and 4.7 times higher compared to the control levels in RTL-W1 cells, 1.5, 1.9, and 3.2 times higher than in T47Dluc cells, and 1.2, 1.4, and 2.2 times higher than in H295R cells following incubation with CNT at 12.50, 25, and 50 mg/L, respectively (Figure 5). The lowest observed effect concentration (LOEC) was 12.50 mg/L for RTL-W1 and T47Dluc cells, with a no observed effect concentration (NOEC) of 6.25 mg/L. For H295R cells, higher LOEC and NOEC were determined amounting to 25 and 12.5 mg CNT/L, respectively.

## Discussion

### Multiwalled carbon nanotubes

In the case of long and stiff CNT, it has been argued that analogous mechanisms to those of other fibrous particles such as asbestos exist [96,97], which may penetrate the lung and persist in the tissue. The biopersistence, large aspect ratio, and fibrogenic character of CNT are important features that may cause adverse health effects. Other mechanisms include hydrophobic contact, through which nanoparticles may interrupt cell membranes, disturbing surface protein receptors [98]. Uptake of nanofibers by human macrophages sized smaller than the length of the nanotubes - a process defined as frustrated phagocytosis - has been shown by backscatter scanning electron microscopy [13]. Overall, nanomaterial size and composition plays a distinct role in the cellular response. In addition, this response is variable between cell types and is likely related to the physiological function of the cell types [95].

However, in our study, flexible multiwalled CNT were investigated for which less concern of their toxic potential has been expressed [99].

### Cytotoxicity

Exposure of RTL-W1, T47Dluc, and H295R cells to 50 mg CNT/L for 24 or 48 h did not induce acute cell toxicity. This is the first study reporting data of cytotoxicity tests with Baytubes using these three cell lines. Several authors have shown that other types of CNT were cytotoxic to different lung epithelial cell lines [100-102], to human astrocyte D384 cells [100], to skin keratinocyte cells, lung cells, T4 lymphocytes [103], and human epidermal keratinocytes [18]. However, in a recent study, Thurnherr et al. [8] also showed that the same type of industrially produced MWCNT had no effect to another cell line. Contradiction to different effects observed in this study and in many other publications might be explained by differences in the CNT material used (metal contaminants, structural defects, size, stiffness, MWCNT vs. SWCNT) and by cell line dependency [8,92]. More likely, positive results are often only due to very high concentrations, which already elicit cytotoxic responses [104,105] or might interfere with the test systems used [106]. The hydrophobic nature of CNT is a general problem when working with these materials not only concerning the generation of stable suspensions that can be applied to the cultures but also for potential interference with the assay due to their high propensity to stick to various molecules or cells [107,108]. For this reason, we used no detergents to prevent MWCNT aggregation during the experiments. The exclusion of such interference with the test systems as well as thorough material characterization is therefore a prerequisite for each study to allow the comparison of results obtained from different researchers [109].

### ROS generation

Main effects of CNT seem to be due to oxidative stress, which triggers inflammation via the activation of oxidative stress-responsive transcription factors [110].

The highest intracellular ROS production could be observed in MWCNT-treated RTL-W1 cells, which was up to five times higher than control levels. A LOEC of 12.5 mg CNT/L was determined. They were followed by MWCNT-treated T47Dluc cells, in which up to three times more ROS was produced compared to the control. The lowest generation of ROS was observed in H295R cells with up to two times higher ROS levels compared to the control level with a LOEC of 25 mg/L.

ROS production can be partially inhibited by metal chelators, indicating that metal components (nickel, iron, yttrium) of CNT are able to contribute to the oxidant response observed [105]. CNT can contain relatively high concentrations of metals as impurities (e.g. 30%), which can contribute to their toxicity. In contrast, purified carbon nanotubes with no bioavailable metals were shown to decrease local oxidative stress development [111], suggesting that similar to fullerenes, ROS may be ’grafted’ at the surface of CNT via radical addition due to their high electron affinity [110]. Barillet and coworkers came also to the conclusion that CNT induced the same level of ROS whatever their length and purity was [92]. They suggested that intracellular ROS production induced by CNT exposure refers to more complex mechanisms than simple redox reactions if we consider the fact that CNT are less accumulated than metal oxide nanoparticles [92].

Ye et al. [102] suggested that ROS and the activation of the redox-sensitive transcription factor NF‒kappaB were involved in upregulation of interleukin‒8 in A549 cells exposed to MWCNT. Yang et al. [112] found that CNT induced significant glutathione depletion, malondialdehyde increase, and ROS generation in a dose‒dependent manner. Pulskamp et al. [50] failed to observe any acute toxicity using the WST-1 assay in cultured rat NR8383 macrophages and A549 cells on viability and inflammation upon incubation with CNT. But they indicated a dose-dependent decrease of the mitochondrial enzyme activity (MTT assay) after 24 h of exposure, similar to the results seen before in other published studies [16,17,113] and detected a dose‒ and time‒dependent increase of intracellular ROS [114]. ROS induction was also observed by exposure to carbon black [115]. Some doubt on the evaluation of MTT toxicity assays were expressed by Wörle-Knirsch et al. [116] because they demonstrated that MTT formazan interacts with CNT interfering with the basic principle of the assay. The authors strongly suggest verifying cytotoxicity data with an independent test system as we did by using different test systems.

A key finding in our study was that ROS generation in three cell lines (RTL-W1, T47Dluc, and H295R) went up in 45 min even in a low dose of incubation group (3.13 mg/L), which was 1.2 times higher than in the controls. Chen et al. [114] assumed that ROS generation came out much earlier than other phenotypes including oxidative stress and cytotoxicity. This might be the reason why other studies in which ROS was measured after more than 4 h exposure to CNT showed inconsistent results [50,117-119]. Several studies [112,120] concluded that cytotoxicity can be attributed to oxidative stress. Interestingly, no cytotoxic effect was found in this study in three different MWCNT-treated cells, although generation of ROS was observed in all cell lines used.

Similar experiments to determine the ROS generation in RTL-W1 cells were performed using multilayer graphene flakes (synthesized by thermal reduction of graphitic oxide at the Federal Institute for Materials and Research and Testing BAM, Berlin) as non-nanomaterial (data not shown). Thereby, same increases of ROS generation were observed up to concentrations of 12.5 mg/L. Whereas, 1.5 times lower increases could be observed for both 25 and 50 mg/L compared to the MWCNT treatment. This lead us to the conclusion that the impurities of metal catalysts (cobalt) are not responsible for the increased production of ROS and such effects may be due to the nanostructure of these materials. Our findings are in accordance with other studies where intracellular ROS generation could be determined by using pristine graphene-treated murine RAW 264.7 macrophages [121], few-layer graphene (3 to 5 layers)-treated PC12 cells [122], and graphene oxide-treated human lung epithelial cells [123] in a time- and dose-dependent manner. However, Creighton et al. [124] showed that graphene-based materials have significant potential to interfere with in vitro toxicity testing methods, such as the H_2_DCF-DA assay, through optical and adsorptive effects at toxicologically relevant doses (less than 10 to 100 mg/L). They could also show that the removal of the nanomaterial by washing can remove optical interferences. Depending on the graphene material, the washing step can lead to accurate data (e.g., for graphene oxide) or to underreporting of ROS as few-layer graphene (3 to 5 layers) adsorbs and quenches the H_2_DCF-DA dye in a manner that depends on surface area [124]. Optical interferences can be excluded for the present study because the cell lines were washed accurately with PBS, but the adsorptive effect is still unclear and may lead to underestimate the production of ROS generation. Still, significant ROS production was observed in all three tested cell lines for the first time after exposure to Baytubes.

### Triclocarban

#### Cytotoxicity

There is very limited information concerning the cytotoxic actions of TCC in mammalian cells, although these actions have been examined, to some extent, in aquatic and terrestrial organisms [125-127]. Morita et al. [126] showed no cell lethality after the incubation of rat thymocytes with TCC at concentrations ranging from 30 to 500 nM for 1 h. The incubation with TCC at concentrations ranging from 10 to 1 μM for 1 h did not affect the viability of rat thymocytes [128]. Another study by Kanbara et al. [129] showed an increase in cell lethality when rat thymocytes were incubated with 10 μM TCC. In the present study, a cytotoxic effect to treated RTL-W1 cells was already observed at concentrations above 4 μM TCC. Both human cell lines (T47Dluc, H295R) showed no cell lethality when exposed up to 1.6 μM TCC. These results are in agreement with the open literature [128,129].

#### Estrogenic activity

As shown in Figure 4, a decrease of luciferase activity in the ER Calux assay was determined after exposure to high TCC concentrations (1.6 μM). Downregulation of estrogen receptors (ER) or other mechanisms of negative feedback may cause this decrease [130]. TCC did not significantly alter the production of E2 in H295R cells up to a concentration of 1.6 μM determined in the ELISA assay.

Ahn et al. [54] observed weak ER activity of TCC at concentrations of 1 and 10 μM. They also found that in the presence of estrogen or testosterone (T), TCC enhanced the actions of these hormones. A cell-based androgen receptor-mediated bioassay with TCC was investigated by Chen et al. [67]. Neither cytotoxicity nor the competition between TCC and testosterone for binding sites could be observed in their studies. However, TCC did amplify testosterone-induced transcriptional activity both in a time- and dose-dependent manner [67]. Altogether, the results suggest that the effects seen with TCC in luciferase-based transactivation assays are due to interference with firefly luciferase, rather than due to causing of the ERα or the androgen receptor (AR) [131]. Similar false positives have been reported in previous high-throughput screens [132]. A recent screen of the NIH Molecular Libraries Small Molecule Repository identified 12% of the 360,864 molecules to be inhibitors of firefly luciferase [133]. In some cases, inhibition paradoxically resulted in an increase of the luminescence signal, probably because of enzyme stabilization [134]. Such a mode of action is also supported by the PubChem Bioassay Database (http://pubchem.ncbi.nlm.nih.gov), which quotes a preliminary EC50 value of 8.9 μM TCC for the inhibition of luciferase.

The focus of the present study was to get more information about the biocide in cell-based assays as well as about interactions of TCC and MWCNT. Our results on the activity of TCC in the ER-responsive cells provide an explanation for the mechanism how chemicals enhance the endocrine-disrupting activity of chemicals [54]. Chemicals acting as endocrine-disrupting compounds (EDC) affect the ER receptor and lead to activation/inhibition of hormone-dependent gene expression [54]. However, EDC may also alter hormone receptor function simply by changing phosphorylation of the receptor (activating him) without the responsible chemical or natural ligand ever binding to the receptor [135].

Clearly, further examinations are required especially the confirmation of our findings in vivo.

Triclocarban at concentrations up to 1.6 μM showed no generation of ROS in three cell lines. Two similar studies suggested the production of reactive oxygen species in rat thymocytes after an incubation time of 1 h to 300 nM or higher concentrations of TCC [126,129]. On the contrary, Fukunaga and coworkers [128] supposed that the same cells recovered the initial loss of cellular glutathione as a biomarker of oxidative stress in the continued presence of 300 nM TCC. Thus, the ability of TCC to generate ROS in human cell lines is still under discussion and further research is required.

### Interaction of MWCNT and TCC

Most reported studies have illustrated that the CNT surface area is an adsorbent for organic chemicals, such as polycyclic aromatic hydrocarbons, phenolic compounds, and endocrine disrupting chemicals [29,136,137]. In the present study, we determined for the first time lower cell toxicity in MWCNT- and TCC-treated H295R cells compared to the cytotoxic potential of TCC alone. Even the antiestrogenic potential of TCC in the ER Calux assay with T47Dluc cells was reduced in the presence of MWCNT compared to the absence of the nanotubes in the whole experimental design. To our knowledge, the influence of MWCNT on the availability of TCC was not examined before. The antimicrobial agent TCC seems to interact with MWCNT resulting in a lower available concentration of TCC in the test medium. This could be proven in the ER Calux assay (Figure 4). Treatment of the cells with higher levels of CNT combined with a lower TCC concentration (0.5% of the nanotubes) did not result in a decrease of luciferase activity compared to same concentrations of the antimicrobial biocide and the mixture of MWCNT and TCC (concentration 1% of that of CNT).

Only few studies have been conducted to understand the adsorption of organic contaminants by CNT [25-27,29,138-140]. A common observation from these studies was that CNT are very strong adsorbents for hydrophobic organic compounds. Possible adsorption mechanisms are the hydrophobic interactions between TCC and CNT or non-covalent π-π electron-donor-acceptor (EDA) interactions [141]. With a log K_OW_ of 4.9 for TCC [59] and considering the strong hydrophobicity and high surface area of carbon nanotubes [142], the hydrophobic effect might be the dominant factor for the adsorption of TCC on the MWCNT. Chen et al. [142] reported that the strong adsorption of polar nitroaromatics, compared to apolar compounds, was due to π-π EDA interactions between the nitroaromatics (π acceptor) and the graphene sheets (π donors) of CNT. An important implication from several of the studies is that electronic polarizability of the aromatic rings on the surface of CNT might considerably enhance adsorption of the organic compounds [25,138-140]. As concluded by Chen and coworkers [142], no studies have been conducted to systematically compare adsorptive interactions between carbon nanotubes and organic compounds with significantly different physical-chemical properties (e.g., polarity, functional groups, etc.). In addition, engineered carbon nanomaterials can vary significantly in shape, size and morphology, and impurity, e.g., metal, amorphous carbon, and O-containing groups, which can further complicate the adsorptive properties of these materials for organic contaminants [142].

## Conclusions

We investigated the cytotoxicity and the endocrine potential of unfunctionalized, flexible MWCNT and their capability to enhance the production of intracellular ROS. TEM analyses revealed the presence of well-dispersed, isolated nanotubes as well as aggregated clusters in our assays. We found that the tested CNT are not toxic to RTL-W1, T47Dluc, and H295R cells. As assumed, we did not find a significant change in luciferase activity in the ER Calux assay with T47Dluc cells nor a significant alteration of E2 production in H295R cells after treatment with MWCNT. Consistent with other studies, this work also shows the generation of ROS by MWCNT. Concentrations (1.6 μM) of the biocide TCC decreased the luciferase activity in ER Calux assays but did not affect the production of E2 in H295R cells in ELISA assays. In mixtures of MWCNT and TCC, the antiestrogen potential of TCC in T47Dluc cells was reduced because the lipophilic biocide adsorbed to the nanotubes resulting in a lower available concentration of TCC in the test medium. More research is needed to better understand the molecular interactions of carbon nanotubes and organic contaminants. In such experiments, the properties of both contaminants, CNT, and pollutants, should be systematically varied.

## Competing interests

The authors declare that they have no competing interests.

## Authors’ contributions

AS (first author) carried out the experimental studies and drafted the manuscript. SM enabled to carry out the in vitro testing of T47Dluc cells and helped to perform one part of the statistical analysis. HH conceived of the study and participated in its design. AS conceived of the study and participated in the sequence alignment. HM participated in the design of the study and helped to perform the statistical analysis and to draft the manuscript. All authors read and approved the final manuscript.

## Authors’ information

AS (first author) is a PhD student at the Institute for Environmental Research at RWTH Aachen University. SM is the head of the working group Endocrine Disruptors at the Institute for Environmental Research at RWTH Aachen University. HH, Prof. Dr. rer. nat., is the director of the Institute for Environmental Research (in cooperation with Prof. Dr. Andreas Schäffer) at RWTH Aachen University, Head of the Department of Ecosystem Analysis, ERASMUS coordinator of the School of Biology, and adjunct professor at Nanjing University (School of the Environment); Dr. Hollert is a member of the Society for Environmental Toxicology and Chemistry, where he is a council member of the SETAC Europe-German Language Branch and a member of the SedNet and Editor-in-Chief ESEU. AS, Prof. Dr. rer. nat., is the director of the Institute for Environmental Research (in cooperation with Prof. Dr. Henner Hollert) at RWTH Aachen University, Chair of Environmental Biology and Chemodynamics, Chair of the board of the Research Institute for Ecosystem analysis and assessment gaiac, adjunct professor Nanjing University (School of the Environment), a member of Society of German Chemistry, Gesellschaft Deutscher Chemiker (GDCh, chair of the board), Society of Environmental Toxicology and Chemistry (SETAC), German Soil Science Society (DBG), expert in the German federal institute for risk assessment, (BfR), and Editorial board Environ. Sci. Pollut. Res. HM, Dr. rer. nat., is the head of the working group Environmental Risk Assessment of Engineered Nanoparticles at the Institute for Environmental Research at RWTH Aachen University.
